# Structural variations in livestock genomes and their associations with phenotypic traits: a review

**DOI:** 10.3389/fvets.2024.1416220

**Published:** 2024-11-12

**Authors:** Yinghui Chen, Muhammad Zahoor Khan, Xinrui Wang, Huili Liang, Wei Ren, Xiyan Kou, Xiaotong Liu, Wenting Chen, Yongdong Peng, Changfa Wang

**Affiliations:** College of Agronomy and Agricultural Engineering Liaocheng University, Liaocheng, China

**Keywords:** structural variations, livestock genome, phenotypic traits, genetic marker, molecular breeding

## Abstract

Genomic structural variation (SV) refers to differences in gene sequences between individuals on a genomic scale. It is widely distributed in the genome, primarily in the form of insertions, deletions, duplications, inversions, and translocations. Due to its characterization by long segments and large coverage, SVs significantly impact the genetic characteristics and production performance of livestock, playing a crucial role in studying breed diversity, biological evolution, and disease correlation. Research on SVs contributes to an enhanced understanding of chromosome function and genetic characteristics and is important for understanding hereditary diseases mechanisms. In this article, we review the concept, classification, main formation mechanisms, detection methods, and advancement of research on SVs in the genomes of cattle, buffalo, equine, sheep, and goats, aiming to reveal the genetic basis of differences in phenotypic traits and adaptive genetic mechanisms through genomic research, which will provide a theoretical basis for better understanding and utilizing the genetic resources of herbivorous livestock.

## 1 Introduction

Structural variation (SV) is a major source of genetic diversity among organisms (1, 2) and is defined as differences in DNA segments greater than 50 base pairs between genomes. The SVs play a crucial role in generating significant phenotypic variability among individuals and facilitating evolutionary adaptations (3–5). These variations arise from various genetic processes, including DNA recombination, replication, and repair mechanisms, which lead to changes in the structural configuration of genomic regions (6, 7). Studies using murine models show that SVs significantly contribute to the genetic heterogeneity observed within species populations (8). Additionally, many of these variations are linked to the development of various human diseases, highlighting their importance in medical genetics (9–11).

The development of genomic variation detection technologies has progressed through several stages, including chromosomal karyotyping, fluorescence *in situ* hybridization (FISH), comparative genomic hybridization (CGH), and microsatellite markers. More recent innovations, such as single nucleotide polymorphism (SNP) chip arrays, array CGH, and high-throughput sequencing technologies, have significantly advanced life sciences. Despite advancements in genomic technologies, accurately detecting SVs in herbivorous livestock remains a significant challenge (1, 12, 13). This difficulty is compounded by lower-quality genome assemblies and incomplete gene annotations, often leading to the misidentification of SVs (1, 13). Understanding SVs is essential in animal genetics, particularly in herbivorous livestock such as cattle and sheep, where SVs are strongly associated with economically significant traits. Substantial evidence suggests that artificial selection has favored advantageous SVs in these species, exemplified by the duplication of the agglutinin signaling protein gene, which is linked to the white coat phenotype in sheep (13). This review provides a comprehensive overview of recent research advancements in the study of SVs—such as copy number variations (CNVs), inversions, and translocations—within the genomes of various livestock species, including cattle, buffalo, equines, sheep, and goats. It also examines how these genomic variants influence key phenotypic traits, such as growth rate, reproductive performance, milk quality, and disease resistance. Through comparative analysis of genomic data across different livestock species, this paper seeks to elucidate the role of SVs in shaping genetic diversity and phenotypic traits, as well as their potential applications in molecular breeding and genetic improvement. Additionally, the review critically assesses current methodologies for detecting and analyzing SVs, highlighting their strengths and limitations in terms of accuracy and resolution. It concludes by proposing future research directions to deepen our understanding of the genetic basis of complex traits in livestock, and to support the sustainable and effective management of livestock genetic resources.

## 2 Classification of SVs

The SVs are diverse and include types such as insertion, deletion, duplication, inversion, and translocation of genomic segments exceeding 50 base pairs (14). Deletion is the most common type of SV, referring to the removal of a segment of DNA sequence from the genome, resulting in a decrease in the number of bases in the genome. Depending on the location of the DNA sequence deletion, it can be categorized as intermediate or terminal deletion (15). Insertion refers to adding a DNA segment within the genome, resulting in a change in the base sequence at that location. Insertion can be classified into two types: general DNA segment insertion, in which the inserted segment usually originates from the genome, and transposon insertion (16). Transposons are a class of DNA sequences that can move or change positions autonomously within the genome. They occupy a significant portion of the genome and are widespread across various organisms’ genomes. Transposon insertion affects the number of gene copies, gene order, the distance between genes, and the regulation of gene expression (17). When transposons are inserted in gene regulatory regions (e.g., promoters, enhancers, etc.), they may interfere with normal regulatory mechanisms, leading to changes in gene expression levels. Transposon insertion is a significant mechanism of genetic variation within the genome, increasing genomic instability, mutation rates, and genetic diversity. Its effects on the genome are diverse and complex and can influence individuals’ genetic characteristics and disease development. This mechanism plays an important role in the evolutionary process (18–20).

Duplication involves the replication of a DNA segment within the genome, resulting in the presence of two or more copies of that segment. Duplicated DNA segments can vary in length, ranging from 10 to millions of base pairs. Duplication can be further categorized into two types: tandem duplications (21) and interspersed segmental duplications. Tandem duplications occur when the duplicated segments are directly linked to form a tandem structure. These duplications usually result from errors or recombination events during DNA replication. Tandem duplications can further be classified as short tandem duplications (22) or long tandem duplications. Short tandem duplications generally range from a few to a few 100 base pairs, while long tandem duplications can comprise several 1,000 base pairs or even more. Interspersed segmental duplications are repetitive segments that occur multiple times in the genome, but they are separated from each other by other DNA sequences (23). Interspersed segmental duplications can involve both duplications of genes and non-gene sequences (24). Duplications contribute to genome evolution by driving the emergence of new genes and isoforms, thereby increasing functional diversity and promoting evolutionary changes. Further, they can predispose individuals to the onset of certain genetic diseases.

**Figure 1:**
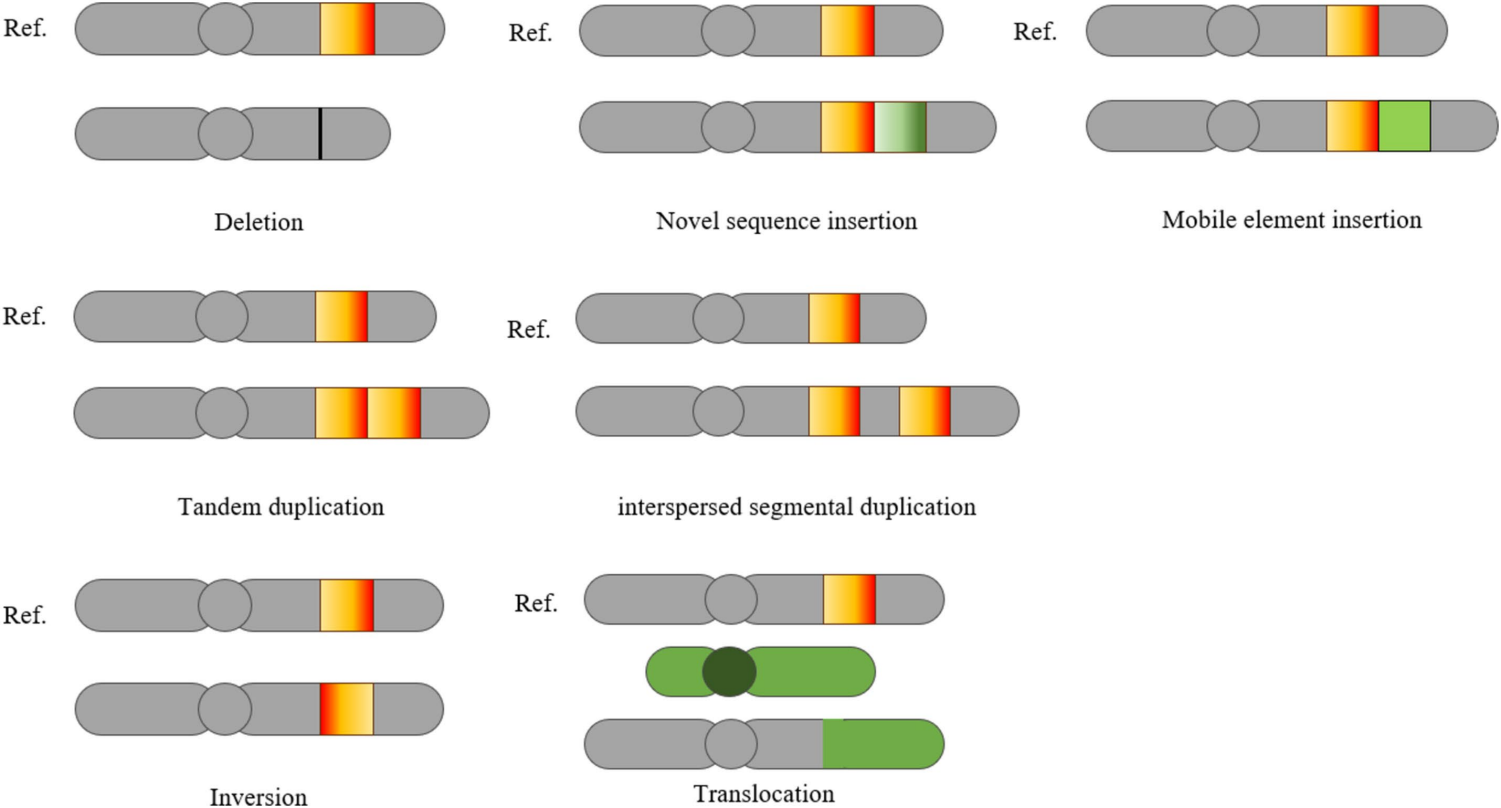
Classification of SVs (27). This schematic illustrates various types of SVs in a test genome compared to the reference genome, including deletions, novel sequence insertions, mobile element insertions, tandem duplication, interspersed segmental duplication, inversions and translocations.

Deletion, insertion, and duplication of genomic fragments longer than 1 kb are classified as CNVs (25). These CNVs are the primary source of genomic SVs (26). The other two categories of SVs, inversion and translocation, involve significant rearrangements, including the relocation of DNA segments between different regions of the genome. SVs can be further divided into balanced and unbalanced events based on the presence of CNVs (14). Unbalanced rearrangements, which include deletions, insertions, and duplications, occur alongside CNVs. In contrast, balanced rearrangements, such as inversions and translocations, involve changes in the order of genomic bases without alterations in CNVs. Distinguishing between these two categories is crucial because the methods used to detect SVs are closely linked to the proportion of genomic sequences that are created or eliminated. In the unbalanced category of SVs, CNVs typically represent a significant portion of the genome. Figure 1 illustrates deletions, insertions, mobile element insertions, tandem repeats, scattered repeats, inversions, and translocations in relation to the reference genome in the test genome (27).

## 3 Mechanisms of SVs formation in the genome

The mechanisms underlying the formation of SVs can arise from various mutational mechanisms (28), which mainly include mobile-element insertion (MEI), fork stalling and template switching (FoSTeS), non-homologous end joining (NHEJ), and non-allelic homologous recombination (NAHR) (13). These mechanisms, including DNA recombination, replication, and repair, are believed to be responsible for structural alterations in DNA segments, resulting in the creation of SVs within the genome.

### 3.1 Mobile elements

The MEIs are discrete segments of genomic DNA that can insert new copies elsewhere in the genome via RNA intermediates (29). In humans, the vast majority of MEIs no longer retain the ability to generate new insertions. However, a few MEIs, mainly from the L1, Alu, and SVA families (30), remain active and capable of generating new insertions. Estimates suggest that approximately 1 in every 12 to 14 live births has a *de novo* MEI (31), making MEIs an endogenous and persistent source of variation in the human genome. They can cause disease by directly disrupting coding sequences or otherwise altering messenger RNAs (mRNAs). For example, the first disease-causing MEI variant identified in humans was the hemizygous variant in F8, which causes hemophilia through loss of function (32). This form of genetic alteration may have significant clinical implications (33).

### 3.2 Fork stalling and template switching

The FoSTeS is a DNA replication-based mechanism that explains complex genomic rearrangements and CNVs (34). In the process of DNA replication, the DNA double helix is unwound to form two replication forks that move in opposite directions along the DNA strand (35). Fork stalling occurs when one or both of the replication forks encounter an obstacle that impedes their progress or DNA damage (36). Template switching is one mechanism that may occur during fork stalling (37), enabling the continuation of the replication process by switching a stalled fork to a nearby intact DNA template (38, 39). FoSTeS is an important mechanism for DNA replication and repair, ensuring that DNA synthesis can proceed even in the presence of barriers or damage (40). Additionally, changes in the site of replication initiation can lead to duplications or deletions.

### 3.3 Non-homologous end joining

The NHEJ mechanism serves as a physiological mechanism utilized by cells to repair DNA double-strand breaks induced by ionizing radiation or reactive oxygen species (41). This repair process typically occurs at low-copy repetitive sequences and is closely related to DNA replication (42, 43). The NHEJ-associated proteins are triggered by double-strand breaks in DNA sequences to facilitate the repair and joining of DNA strands (44). Initially, end repair replaces the lost nucleotides at the double-strand break, after which DNA ligase joins the broken DNA fragments together. The joining of segments from different chromosomes can lead to the duplication or deletion of sequences (13).

### 3.4 Non-allelic homologous recombination

**Figure 2:**
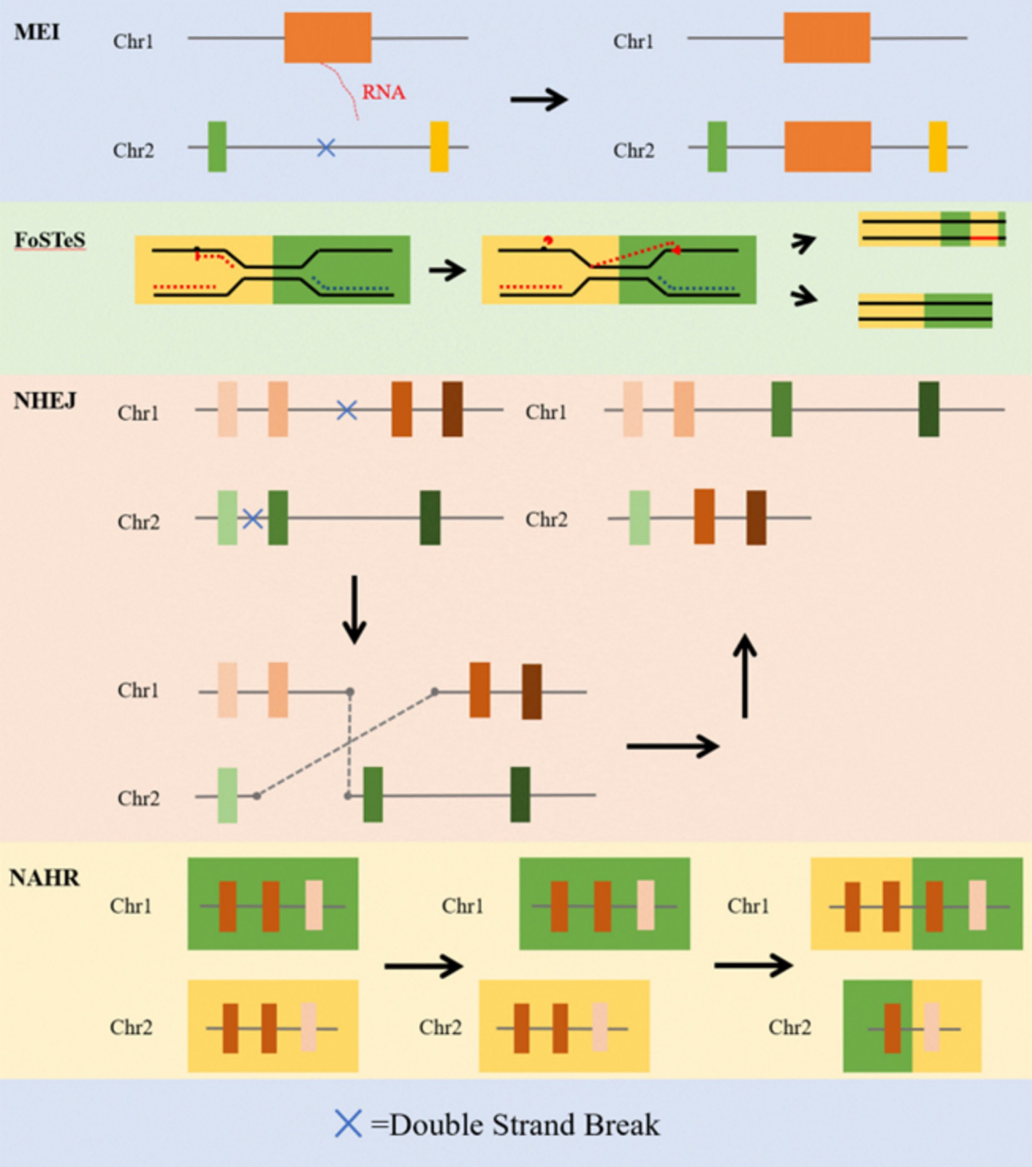
The main formation mechanisms of SV (12). The schematic depicts the process of the main formation mechanisms of SV, including mobile-element insertion (MEI), fork stalling and template switching (FoSTeS), non-homologous end joining (NHEJ) and non-allelic homologous recombination (NAHR).

The NAHR produces SVs when a genomic segment exhibits high sequence similarity to a non-allelic locus (13). This recombination can lead to the duplication of similar sites on one chromosome and the corresponding deletion of sites on the other chromosome. The NAHR commonly occurs during meiosis and mitosis because two regions with similar sequences on non-homologous chromosomes are susceptible to recombination (45–47). This process can disrupt genetic information, potentially resulting in abnormal phenotypes. Duplicate elements are often located at the breakpoints of NAHR events that are associated with cancer and various genetic disorders (48–51). Additionally, the process of crossover between sister chromatids may result in the addition or loss of DNA segments, leading to duplications, deletions, and inversions of chromosomal segments (Figure 2).

## 4 Detection methods for SVs in genome

The unbalanced events are typically detected through the loss or gain of genomic sequence (referred to as “read depth” or RD) (52, 53) or the array probe signal intensity (54, 55) in the affected region when compared to the reference genome. There is a need to identify sequence breakpoints for the detection of balanced events, and methods designed to identify unbalanced SVs from array and sequence data are more sophisticated than those focusing on balanced events (27). Balanced SVs, such as inversions and chromosomal translocations, have the potential to impact the phenotype of an organism but remain particularly challenging to identify as *de novo* events due to their negligible impact on gene copy numbers (56). Inversions are almost undetectable, with viable detection methods limited to PCR (57) and sequencing (6). Specialized sequencing methods utilizing bipartite sequence data (referred to as “read pairs” or RPs) have subsequently been developed to detect these inversions.

Sanger sequencing technologies offer high accuracy but have low throughput. In contrast, next-generation sequencing technologies excel in cost and throughput, although they have shorter read lengths and higher error rates. Third-generation sequencing technologies provide significant advantages in read lengths but are associated with higher error rates and require more complex data processing (58–61). Several bioinformatics technologies, including RNA-Seq, ChIP-Seq, FAIRE-Seq, ChIA-PET, and Hi-C, utilize next-generation sequencing (NGS), a technology named for its significantly higher throughput compared to first-generation sequencing (58). Presently, Illumina sequencing technology (62) is the most commonly employed, capable of generating 100 of gigabytes or even several terabytes of sequencing data within a matter of hours, thus satisfying the throughput demands of high-throughput sequencing, while ensuring the accuracy of its sequencing. The fundamental principle of Illumina sequencing involves the reversible termination of fluorescently labeled dNTP to facilitate synthesis-while-sequencing (63).

Third-generation sequencing technology, known as single-molecule real-time sequencing technology or *de novo* sequencing technology, distinguishes itself from previous generations by its primary feature of single-molecule sequencing without the need for PCR amplification, enabling individual sequencing of each DNA molecule. While second-generation short-read sequencing technology currently dominates the sequencing market, the third-generation technology has gained momentum in recent years and has been applied to genome sequencing, methylation research, mutation identification, and other research fields. The primary third-generation sequencing technologies are nanopore electrical signal sequencing and single-molecule fluorescence signal sequencing. Nanopore electrical signal sequencing encompasses single-molecule nanopore DNA sequencing by Oxford Nanopore Technologies (ONT). Single-molecule fluorescence signal sequencing comprises single molecule real-time (SMRT) technology by Pacific Biosciences (PacBio). Among these, the cornerstone of third-generation sequencing is the Nanopore sequencing technology developed by Oxford Nanopore (64, 65). The principle behind Nanopore sequencing involves using a nanopore covalently bound with molecular junctions inside the pore, with nanopore proteins immobilized on a resistive membrane. Kinetic proteins are then used to pull the nucleic acids through the nanopore. As the nucleic acid moves through the nanopore, it causes a change in charge, resulting in a change in the electrical current across the resistive membrane. Due to the small diameter of the nanopore, only a single nucleic acid polymer can pass through, and the charged nature of individual ATCG bases causes varying interference to the current, enabling real-time monitoring and decoding of current signals to determine the base sequence and achieve sequencing (66). Technologies for third-generation sequencing (TGS) can produce read lengths up to 10 of kilobase pairs (kb) or longer, allowing for detailed characterization of complex genomic regions, such as duplications, which are difficult to accurately analyze with short-read sequencing methods. Due to its long-read capability, TGS improves the accuracy of SV breakpoint and type identification, essential for understanding the biological impact of these variations (59). Various methods and tools for SV detection based on third-generation sequencing include PanPop (67), cuteSV (68, 69), cuteSV2 (70), DeBreak (71, 72), DELLY (73–75), and SVision (76).

Optical genome mapping (OGM) is a genome analysis technique that visualizes structural variation by directly imaging ultra-long DNA molecules (77, 78). This OGM technology employs restriction endonucleases and fluorescent markers for labeling DNA, followed by high-resolution imaging to capture the labeling patterns. These patterns reveal structural details across the genome, such as fragment size, position, and relationships. In addition, OGM technology provides advantages in high resolution (79, 80), ultra-long read lengths (79), sensitivity, specificity (80), and PCR-free analysis. However, OGM technology remains relatively new, with limitations in maturity, higher costs, and longer processing times (79). Genome-wide association study (GWAS) involves the detection of genome-wide polymorphisms in multiple individuals to obtain their respective genotypes (81). Subsequently, statistical analysis at the population level is carried out to examine the relationship between the obtained genotypes and the corresponding phenotypes. Genetic variants most likely to influence the trait are filtered based on statistical significance, followed by the identification of genes associated with these trait variants (82). GWAS analysis utilizes two kinds of data: genotypic data, usually in the form of a vcf file, and phenotypic data, typically in the format of a txt file containing sample names and their corresponding trait lists. These genetic markers, derived from these data, can subsequently be utilized for the development of breeding-related test chips or for their value in medical diagnosis (83). While the principles underpinning GWAS for plant and animal breeding and human disease treatment do not significantly differ, the practical applications vary considerably. Consequently, the GWAS process for one species may not be directly transferable to another species (84). By using GWAS analysis, a study identifies structural variants, including insertions, deletions, inversions, and translocations, by comparing sequencing data to a reference genome with software tools such as cuteSV (68, 69), BreakDancer (85), Pindel (86, 87), and SVMerge (88).

## 5 SVs in livestock genome and their association with phenotypic traits

The importance of genetic variations has been extensively discussed in livestock animals (89, 90). These variations can impact gene expression and regulatory mechanisms, influencing phenotypic traits such as growth rate, milk production, disease resistance, and fertility in various livestock (91–96). Understanding SVs in livestock genomes enhances our ability to predict and manipulate traits, contributing to advancements in agriculture and food security and improved breeding programs and more efficient livestock production. Below, we have discussed the research development on SVs in livestock genomes including cattle, buffalo, equine, sheep and goat.

### 5.1 SVs in cattle genome and their association with phenotypic traits

Genomic SVs represent an important source of genetic variation in cattle genomes and are commonly linked to phenotypic expressions (97–108). Substantial progress has been made in understanding SVs concerning cattle breed genetic characteristics (109–118) and their associations with essential phenotypes, including feed intake (119), growth traits (120), milk production (121–123), disease resistance or susceptibility (124–127), reproductive health (128–130), coat color patterning (57, 131–133) and environmental adaptability (134, 135) in cattle.

In studies focusing on growth traits, research on the *EIF4A2* gene in four cattle breeds—Qinchuan, Yunling, Pinang, and Jiaxian—demonstrated that the *EIF4A2-CNV* gene significantly influenced hip width and rump length in Qinchuan cattle, heart girth, chest depth, and rump length in Yunling cattle, and hip width in Pinang cattle (136). No significant effect on hip width was observed in Jiaxian cattle, suggesting the potential of *EIF4A2* gene SVs as molecular markers in yellow cattle breeding, with implications for enhancing the selection of superior beef breeds (136). Consistently, studies analyzing GWAS data for CNVs and body growth traits in beef cattle have focused on the Nellore breed (120, 137). Using data from over 700,000 SNP probes in 2,230 cattle, CNVs such as EPHB3-CNV98, *COL26A*-CNV121, *GBP6*-CNV204, *ZNF280B*-CNV96, and *TSPY*-CNV99 were found to be significantly associated with growth traits in the Nellore breed (137). In addition, a CNV100 overlaps with the *KCNJ12* gene, was observed to be a key candidate for muscle development (137). Accordingly, a study investigated CNVR in the Brazilian Gir dairy cattle genome, focusing on traits relevant to tropical breeding conditions. By analyzing sequencing and SNP genotyping data from 38 animals, 48 high-confidence CNVR were identified. These regions were associated with genes linked to traits like environmental adaptation, immune response, and reproduction (138).

In relation to milk production, 24,908 high-quality SVs were identified in a cohort of 478 Holstein and Jersey cows through whole-genome sequencing. An interpolation technique estimated 4,489 SVs with an R^2^ greater than 0.5 in 35,568 Holstein and Jersey cows, utilizing two pipelines: FImpute and Quille2.3-Minimac 3 (139). Their findings further revealed that SVs typically explained less than 10% of the phenotypic variation in key dairy traits, with four SVs significantly associated with these traits (139). Concerning genetic characteristics, Talenti et al. employed optical mapping to construct a high-quality SV database among various cattle breeds from different geographical regions, thereby advancing research on SVs in cattle. Specifically, Bionano optical mapping data at 100X coverage were generated for 18 cattle from nine ancestral lines across three continents and two subspecies. This study identified 13,457 SVs, with 1,200 of which overlapped coding regions (140). In the context of disease resistance and climate adaptation, a comparison of chromosome-scale genome assemblies in two cattle genealogies identified 123,898 non-redundant SVs. Functional studies suggested that a 108 bp exon insertion in the sialophorin (*SPN*) gene may affect macrophage uptake of *Mycobacterium tuberculosis,* contributing to the reduced susceptibility of Hainan yellow cattle to bovine tuberculosis (141). In line other studies also reported the association of CNVs with mastitis resistance in Dutch Holstein cattle (142), hoof and reproductive health in Canadian Holstein cattle (143, 144). In addition, research developed a novel SV detection pipeline, identifying millions of deletions, inversions, and duplicated regions in the cattle genome. A deletion variant in the APPL2 gene’s first exon was found to impact gene expression related to immune response, metabolism, and other functions, highlighting its role in selective adaptation across different regions. (145). Furthermore, a study focuses on mapping expression and splicing quantitative trait loci (e/sQTL) to understand phenotypic variability in cattle (146). The researchers created a pangenome using 16 HiFi haplotype-resolved cattle assemblies and genotyped 307 short-read samples, identifying over 21 million small and 43,000 structural variants. They validated 85% of structural variants and mapped e/sQTLs in 117 cattle with testis transcriptome data, identifying 92 structural variants as causal candidates for eQTL and 73 for sQTL. Transposable elements were found to be key contributors to expression and splicing variation. Despite strong linkage disequilibrium between small and structural variants, only 28 additional eQTL and 17 sQTL were discovered (146).

### 5.2 SVs in buffalo genome and their association with phenotypic traits

The SVs in the buffalo genome and their association with phenotypic traits have been a subject of increasing interest in recent studies. For instance, Ahmad et al. (147) employed a coverage-based approach to generate high-resolution CNV maps of six major buffalo breeds globally using whole-genome resequencing data. By analyzing data at two sequencing coverage levels, 10X and 30X, they detected a total of 14,368 CNVs at 10X coverage and 127,222 CNVs at 30X coverage, with deletions outnumbering duplications in all breeds. At 10X coverage, the Murrah breed exhibited the highest number of CNVs, while the Surti breed had the lowest. Conversely, at 30X coverage, the Pandharpuri breed had the highest CNV count, while the Surti breed retained the lowest (147). Comparison of CNV profiles across these breeds highlighted evolutionary divergences among major buffalo breeds worldwide. This study enhances our understanding of SV in buffaloes and holds promise for applications in selective breeding and genetic improvement efforts (147). In another study, Li et al. characterized genomic differences between the water buffalo genome and the well-studied *Bos taurus* cattle genome. By comparing whole-genome sequencing datasets of 14 river buffaloes to the cattle reference genome, they identified 13,444 deleted CNV regions and 11,050 merged MEI events located upstream of annotated cattle genes. These findings provide essential data for the functional annotation of genes that may be linked to phenotypic differences between cattle and buffalo, laying the groundwork for future genomic analyses (148). Further advancing the understanding of buffalo genomics, Wang et al. reported a chromosome-level genome assembly with a 72.2 Mb contig N50 and a high-resolution recombination map for male buffalo. Their study revealed that transposable elements (TEs) and SVs have potentially contributed to buffalo evolution by influencing neighboring gene expression. Notably, the pseudoautosomal region (PAR) of the Y chromosome was found to be under strong purifying selection. Additionally, two distinct recombination hotspots were identified on chromosome 8, near genes associated with tooth development, which may enhance buffalo adaptation to low-quality feeds. Additionally, they found that the TE subfamily SINE/tRNAs may play a role in driving recombination into SVs, offering important insights into buffalo genome evolution and adaptation (149). Moreover, Strillacci et al. performed a genome-wide CNV scan on 361 buffaloes from three Iranian river breeds (Azeri, Khuzestani, and Mazandarani), detecting 9,550 CNVs and 302 CNV regions (CNVRs), which encompassed 1.97% of the buffalo genome. Notably, 22 CNVRs were common across all breeds, and 409 genes mapped to CNVRs were linked to traits such as morphology, health, milk production, meat quality, and reproduction, as annotated in the Bovine Genome Database. These results advance our understanding of the natural adaptations and recent environmental pressures faced by buffaloes, particularly in relation to milk production, their primary food source (150). In addition, Li et al. used comparative genomic and transcriptomic analyses to highlight significant structural genomic differences between river buffalo and taurine cattle. These differences may hold important implications for the biology, adaptation, and evolution of the two species, providing a comprehensive understanding of the river buffalo genome. As a result, this research offers a robust framework for future investigations into genetic improvement and disease resistance in buffaloes (151). Deng et al. further expanded the knowledge base by resequencing the genomes of 387 buffaloes from 29 Asian breeds, including river, swamp, and crossbred buffaloes. They identified 36,548 CNVs through the CNV caller, covering 133.29 Mb of the buffalo genome, alongside 2,100 CNVRs, of which 1,993 were shared among the studied breeds. Population differentiation analysis using Vst identified 11 genes significantly differentiated across buffalo breeds, many of which were associated with milk production traits. Furthermore, expression quantitative trait loci (eQTL) analysis revealed differentially expressed CNVR-derived genes (DECGs) linked to milk production. Through a GWAS analysis, three CNVRs were found to be significantly associated with peak milk production. Collectively, this study provides comprehensive genomic insights into buffalo populations, identifying candidate genes for milk production traits that can inform genetic breeding programs aimed at enhancing milk yield and quality in buffaloes (152).

### 5.3 SVs in sheep and goat genomes and their association with phenotypic traits

SVs in the genomes of sheep and goats have emerged as key contributors to understanding phenotypic traits, especially regarding growth, genetic characteristics, reproduction, and adaptation in sheep and goat (26, 153–165). For example, Jiang et al. examined growth traits by analyzing the CNVs of the *Src Homology 2 Domain Containing E* (*SHE*) gene in 750 sheep specimens, including Chaka sheep, Hu sheep, small tail Han sheep, and large tail Han sheep. The study revealed a 2000 bp CNV in the *SHE* gene. This CNV was associated with traits such as body length, chest width, heart girth, and height at the withers. The study also highlighted breed differences, with deletions in *SHE* more frequents in Chaka and Hu sheep than in small and large tail Han sheep. The researchers concluded that the CNV of the *SHE* gene may be a critical factor in sheep molecular breeding, offering insights for improving economic traits through breeding practices (166). Similarly, CNVs have been identified as playing a significant role in goat reproduction. For instance, in a study on highly fertile dairy goats, researchers found that *PRP 1* and *PRP 6*, both associated with the prolactin (PRL) signaling pathway, had repeated copy numbers in highly fertile goats (167). *PRP 1* copy numbers were repeated three times, while *PRP 6* copy numbers were repeated six times in the high fertility group, contrasting with the normal copy numbers in low fertility goats. These results suggest that the copy number repeats might influence the expression pattern of *PRP 1* and *PRP 6*, though further research is required to clarify the underlying mechanisms (168). In another study, Li et al. performed high-depth resequencing on 16 wild Asian mouflon sheep, 172 local breed specimens, and 60 individuals from various sheep breeds across Asia, Europe, Africa, and Middle East (169). Their analysis identified candidate genes associated with domestication traits like tail fat, horn type, ear size, and other production traits such as wool, milk and meat. This research offered crucial genomic resources for sheep genetics and holds promise for future molecular-assisted breeding efforts (169). Furthermore, a detailed catalog of SVs in sheep was developed using high-quality *de novo* assemblies, revealing a 168 bp insertion segment in the 5′ untranslated region (5’ UTR) of the Homeobox B13 *(HOXB13)* gene (170). This specific mutation was linked to the long-tailed trait in sheep through a combination of GWAS and gene expression analyses (170). Additionally, Shi et al. conducted an in-depth analysis of CNVs in Tibetan sheep, comparing local Oula sheep with synthetic Panou sheep, and identified 60,429 CNV events, including 368 differential CNV regions. Of particular interest, the duplication of the *ABCB1* gene was suggested as a key factor aiding Panou sheep in adapting to high-altitude environments (171). This research provided an extensive CNV map of Tibetan sheep, serving as a valuable genomic resource for future breeding initiatives (171). Consistently, another study identified a CNVR on chromosome 6, which encompasses the *HGFAC* and *LRPAP1* genes—both of which are associated with fat deposition and environmental adaptability in Iranian fat-tailed breeds (Baluchi and Lori-Bakhtiari sheep) as well as thin-tailed breeds (Zel sheep) (172).

In a large-scale genomic study, Liu et al. (26) identified 6,286 potential CNVs across 1,023 samples from 50 goat breeds, covering approximately 262 Mb or 8.96% of the goat genome. Several noteworthy CNV-overlapping genes, including EDNRA, ADAMTS20, ASIP, and DGAT1, were found to be involved in local adaptations such as coat color, muscle development, metabolic processes, and bone formation. This comprehensive CNV map provides new insights into the functional annotation of the goat genome (26). The findings highlight the significant role of SVs, particularly CNVs, in influencing phenotypic variation, breed-specific traits, and local adaptations in sheep and goats. Moreover, these results serve as a crucial genomic resource for future breeding programs and genetic improvement strategies in these species.

### 5.4 SVs in equine (horses and donkeys) genomes and their association with phenotypic traits

SVs in equine genomes, particularly in horses and donkeys, have been the focus of recent research due to their potential impact on phenotypic traits. In particular, advances in genome sequencing technologies have facilitated a more detailed exploration of SVs, revealing their associations with traits such as fertility, environmental adaptability, and high-altitude survival. The rapid progress in science and technology has spurred significant growth in the horse and donkey industries, contributing significantly to the field of animal husbandry (173). Consequently, research into SVs within these animals’ genomes holds substantial importance. Equine genome has been investigated for structural variations and their consequent correlation with phenotypic traits (174–178). Similarly, the copy number of five genes located on the donkey’s Y chromosome—*CUL4BY, ETSTY1, ETSTY4, ETSTY5,* and *SRY*—was quantified, revealing variability in their copy numbers, which offers essential genetic data for future donkey research (179). Additionally, a chromosome-level *Equus kiang* genome was assembled using Hi-C sequencing, leading to the identification of SVs potentially linked to high-altitude adaptation, specifically through species-specific insertions and deletions in genes such as *PIK3CB* and *AKT,* which are implicated in hypoxia-related pathways (180). Further research identified that while moderate expression levels of equine *CUL4BY* were found across various tissues, *ETSTY1, ETSTY4,* and *ETSTY5* showed exclusive expression in the testis of horses, though the status of the equine *SRY* gene as a single-copy gene remains debatable (181). For instance, a study using whole genomes from six diverse horse breeds (Mangalarga Marchador, Percheron, Arabian, Native Mongolian Chakouyi, Tennessee Walking Horse and American Miniature) were sequenced and mapped to the EquCab3.0 genome, generating 1.3 billion reads with coverage between 15x to 24x per horse. After rigorous filtration, they reported 1,923,693 Insertions/Deletions (INDELs), 1,540 CNVs, and 3,321 SVs per horse and functionally annotated. Key genes associated with size variation, such as *LCORL* (in all horses), *ZFAT* (in Arabian, American Miniature, and Percheron), and *ANKRD1* (in Native Mongolian Chakouyi), were detected. Additionally, a copy number variation in the *Latherin* gene, linked to thermoregulation by sweating, was found (182). A genome-wide map of CNVs in Chinese local horses identified candidate genes overlapping with CNVRs in Jinjiang horses, uncovering genes linked to hemoglobin binding. This discovery is of particular interest, as it suggests a role in the adaptation of Jinjiang horses to high-temperature and high-humidity environments, providing key insights into the genetic mechanisms underlying equine adaptation to diverse environmental conditions (183). Consequently, Castaneda et al. (184) analyzed CNVs in horse Y chromosome genes using digital droplet PCR, examining 209 normal males, 73 XY horses with disorders of sex development and/or infertility and 5 Przewalski’s horses and 2 kulans. TSPY showed high variability, while *SRY* copy variations linked to *RBMY* may cause XY disorders of sex development and/or infertility. The CNVs in *TSPY* and *ETSTY2* differed in cryptorchid cases but not in infertility. They suggested further research to refine Y chromosome assembly and its reproductive implications (184) (Table 1).

**Table 1:** Summary of SVs associated with phenotypic traits in herbivorous livestock.

Genes (SVs)	Phenotypes	Biological effect	Species	References
*EIF4A2*	Growth traits	Hip width; rump length; heart girth; chest depth	Cattle	(136)
Deletion in Chr5 93,504,218 bp-93505234 bp, Chr6 87,209,737 bp-87211122 bp, Chr14 1,299,687 bp-1299831 bp, Chr20 28,914,471 bp-28915027 bp	Production traits	Milk, fat, protein yield, fertility and overall type		(139)
316 bp deletion within the intron of *DDX58*	Diseases and adaptations	Climate adaptation, Epidermal differentiation, skin barrier and bovine tuberculosis resistance		(141)
*APPL2*	Diseases and adaptations	Immune response, taste function, cell proliferation, and glucose metabolism		(145)
*RXFP4*	Metabolism	Feed efficiency and feed intake-related traits.		(186)
*LEPR*	Growth traits	Body weight, height and length		(187)
*KIT*	Genetic characteristic	Coat color		(188)
*ZEB2*	Growth traits	Growth, weight traits, and horn ontogenesis	Buffalo	(147)
*STK17 A*	Diseases and adaptations	Involved in apoptosis, which has functions in immune response and disease resistance		(147)
*OR10J5*, *OR10J1*, *OR10J4*, *OR10J3*	Adaptations	Perception of chemical stimuli		(147)
*IBSP*, *SPP1*, *MEPE*	Adaptations	Promoting the formation and mineralization of dentin		(149)
*ABCC8*, *USH1C*, *MYOD1*, *OTOG*, *KCNC1*, *SERGEF*	Production traits	May potentially affecting meat production		(150)
*PLXNA2*		Associated with cattle temperament		(150)
*GLYAT*	Adaptations	Adaptation to tropical environments		(151)
*FTH1, MYPN, NEXN*, *TMOD1*	Production traits	Meat quality		(151)
*RAPH1, DAAM1, U6, TRNAC-ACA, MEX3C, ALDH4A1, MIGA1, SMAD4, PHKA2, COL4A1, ZNF407*	Production traits	Milk production		(152)
*ACTR3, TAS1R2, PBRM1, GNA12*	Growth traits	Carcass and body traits		(152)
*USP33*	Diseases	Bovine respiratory disease susceptibility		(152)
*FNIP2*	Reproductive traits	Reproductive traits		(152)
*SHE*	Growth traits	Body length, cannon bone circumference, heart girth, chest width, and height at the withers	Sheep	(166)
	Production and body size traits	Domestication, tail fat, horn type, ear size, reproductive traits, wool production, milk production, meat production		(170)
168 bp insertion segment in the 5’ UTR of the *HOXB13* gene		Long-tailed trait		(171)
*PPP3CA*, *SSTR 1*		Aging and carcass weight		(172)
The duplication of the *ABCB1* gene	Adaptations	Adaptation to the plateau environment		(172)
*PRP 1*, *PRP 6*	Reproductive traits	Fertility	Goat	(167, 168)
*EDNRA*, *ADAMTS20*, *ASIP*, *KDM5B*, *ADAM8*, *DGAT1*, *CHRNB1*, *CLCN7*, *EXOSC4*	Genetic traits	Coat color, muscle development, metabolic processes, bone sclerosis, and embryonic development		(26)
*CUL4BY*, *ETSTY1*, *ETSTY4*, *ETSTY5*, *SRY*		Sperm quality traits	Donkey	(180)
*PIK3CB* and *AKT*, with lengths of 3,258 bp in the exonic region and 189 bp in the intronic region	Adaptations	High-altitude adaptation	*Equus kiang*	(181)
*CUL4BY*		Moderate expression levels in the testis, heart, spleen, and kidney	Horses	(182)
*ETSTY1*, *ETSTY4*, *ETSTY5*		Exclusively expressed in the testis		
*HSPA1A*, *NFKBIA*, *SOCS4, IL-6*	Adaptations	Adaptation to high-temperature and high-humidity environments		(183)

## 6 Limitations and challenges of SVs in herbivorous livestock

The research on SVs is very important in the field of genomics, which involves variations in DNA sequences such as deletions, insertions, duplications, inversions, and translocations within large segments of the genome. These variations have significant implications for gene expression regulation, disease occurrence, and species evolution. However, SVs research faces several limitations and challenges (13), including constraints of sequencing technology, algorithmic and software issues, sample and population coverage, difficulties in functional verification, lack of phenotypic data, environmental and genetic interactions, challenges for breeding applications, technology costs and accessibility, as well as data sharing and standardization.

Conventional sequencing technologies, such as short-read sequencing, have limitations in detecting structural variants in large fragments, as they struggle to capture DNA sequence changes over long distances. While third-generation sequencing technologies, such as PacBio and Nanopore, offer longer read lengths that can improve the accuracy of structural variant detection, they are also more costly and complex to analyze. SV detection requires complex bioinformatics algorithms that must accurately recognize and distinguish between different types of structural variants. Existing algorithms still struggle with highly repetitive sequence regions, which can lead to false-positive or false-negative results.

The population structure of livestock is complex, with significant differences in genetic background between species and populations. This complexity requires researchers to consider the representativeness and diversity of samples in their analyses, as well as how to verify the biological significance of SVs in different populations. Although computational methods can predict SVs, these predictions usually need to be validated through experimental methods such as PCR and FISH, increasing the complexity and cost of the study. Research into the associations between SVs and production traits in livestock requires large amounts of phenotypic data; however, the collection and integration of these data can be time-consuming and costly.

Experimentation on domestic animals must adhere to strict ethical and welfare standards, which may limit certain types of research. Additionally, production traits in herbivorous livestock are influenced not only by genetic factors but also by environmental ones. Understanding these reciprocal effects is crucial for unraveling the biological functions of SVs. Translating findings on structural variants into practical breeding strategies presents many challenges, including the assessment of variant pathogenicity, genetic counseling, and the development of personalized treatment protocols. Although the cost of sequencing technology is decreasing, accessing and analyzing high-throughput sequencing data remains a financial burden for many researchers. The sharing and standardization of SV data are essential for facilitating global research collaboration and improving research efficiency, yet there is a lack of uniform data formats and sharing platforms.

To overcome these limitations and challenges, researchers need to develop new sequencing technologies, improve algorithms, increase computational power, and promote data sharing and standardization. For example, the SVision (76) and SVision-pro (185) algorithms developed by Prof. Kai Ye’s team enhance the accuracy and reduce the false-positive rate in SV detection by transforming the sequence problem into a variation instance segmentation problem in the image space. These efforts will help improve our understanding of SVs in herbivorous livestock and ultimately enhance their production and health.

## 7 Conclusion

Altogether, we concluded that SVs are a significant source of genetic diversity among individuals. The advent of high-throughput sequencing technology has made genome sequencing of herbivorous livestock more accessible and cost-effective. By comparing genome sequences across different species or individuals, we can identify genomic SVs associated with specific traits. These variations may be linked to important characteristics such as growth rate, reproductive ability, disease resistance, and environmental adaptation. Understanding how these variants affect gene function and expression can help clarify the relationship between genomic SVs and the traits of herbivorous livestock, as well as inform more effective conservation and breeding strategies. Additionally, this research can reveal the evolutionary history and relationships of these animals, enhancing our understanding of their origin and evolution. Both domestic and international studies on genomic SVs in livestock have progressed rapidly, offering valuable insights into the genetic traits, evolutionary history, and population structure of herbivorous livestock.

Future investigations into SVs in livestock genomes should prioritize the development of more efficient and cost-effective long-read sequencing technologies. Such advancements will enhance the accuracy of SV detection, enabling comprehensive studies across large and genetically diverse populations. Additionally, there is a critical need for improved bioinformatics algorithms designed to manage the complexity inherent in genomic regions. These algorithms should aim to minimize sequencing errors and accurately differentiate functional SVs from neutral variations, thereby increasing the reliability of genomic analyses. The expansion of population-scale datasets is essential, along with the establishment of robust data-sharing platforms. These initiatives will facilitate cross-species analyses and comparative genomics, thereby deepening our understanding of SVs across various livestock species. Furthermore, the integration of multi-omics approaches, including transcriptomics and epigenomics, is vital for linking SVs to phenotypic traits. This integration will provide valuable insights into the functional roles of SVs within the context of livestock genetics. Collaborative efforts toward data standardization and the establishment of ethical frameworks are crucial for advancing research and its practical applications in livestock breeding and management.
